# 

**Published:** 1881-05

**Authors:** 


### THE AMERICAN JOURNAL OF DENTAL SCIENCE,

EDITED BY

F. J. S. GORGAS, M. D., D. D. S.,

AND

JAMES B. HODGKIN, D. D. S.

S.G.O.L. INDEXED

VOL. 15.—THIRD SERIES.

SURGEON GEN'L'S OFFICE LIBRARY. 86610

BALTIMORE:

SNOWDEN & COWMAN.

LONDON:

TRUBNER & CO., 60 PATERNOSTER ROW.

1881 —1882.

### Index to Volume XV.---Third Series.

A Few Words about Plastic Materials, - - - 23
A Case of Fracture of the Teeth Causing Severe Symptoms, 47
Alabama Dental Association, - - - 88, 135
Alcohol a Stimulant or Narcotic, - - - - 48
Another Journal, - - - - - - 137
Anæsthetics, Relative Action of - - - - 18
Anæsthesia—Nitrous Oxide, - - - - - 49
American Dental Association, - - - - 133
American Dental Convention, - - - - - 135
Apparent Insusceptibility to Influence of Nitrous Oxide, 143
Alcohol as a Stimulant, - - - - - - 239
Antiseptic Treatment of Alveolar Abscess, - - 289
Alveolar Abscesses, Treatment of - - - 289, 302
A Good Gold Foil, - - - - - - 377
A Discovery Rivalling Dr. Jenner's, - - - 426
About Appointment Books, - - - - - 428
Artificial Milk, - - - - - - 429
A Two-Thousand Dollar Tooth, - - - - 430
About Raising Healthy Women, - - - - 432
A Third Set of Teeth, - - - - - 432
A Tooth in the Bronchus Causing Pneumonia, - - 522
Annealing Gold, - - - - - - 525
An Anæsthetic Car, - - - - - - 525
Atrophy of the Teeth, - - - - - - 468
Alveolar Ulceration as Distinguished
from Alveolar Abscess, - - - - 145
A New Source of Electricity, - - - - 186
Analyzing Metals by Electrolysis, - - - 192
Baltimore College of Dental Surgery,
New Building, - - - - - - 88
Baltimore College of Dental Surgery,
Commencement of - - - - - 470, 518
Blake, Chas. A. - - - - - - - 466
Blount, A. A., M. D., D. S., - - - - - 416
Bonwill, W. G. A., D. D. S., - - - - 111
Bonwill's Electro-Magnetic Mallet, - - - 135, 163
Bleaching Teeth, - - - - - 82, 123
Bowne, Edward H. D. D. S., - - - - - 319
Bogus Diploma Traffic, - - - - - 34
Bouton, A. G., D. D. S., - - - - - 366

Bromide of Ethyl as an Anæsthetic, - - - 238
Bonwill's Method of Restoring Crowns upon Roots, - 423
BIBLIOGRAPHICAL.
Plastics and Plastic Fillings, - - - 41
Illustrated Scientific News, - - - - 42
Ohio State Journal of Dental Science, - - 42
Coles' Deformities of the Mouth, - - - 383
The American Agriculturist, - - - 383
Benedikt on Brains of Criminals, - - - 383
The Scientific American, - - - 384
Physicians' Visiting List for 1882, - - - 384
The Sanitarian, - - - - - 384
Sea Sickness, - - - - 384
Opium Habit and Alcoholism, - - - 473
New England Journal of Dentistry, - - - 474
The Prevention of Syphilis, - - - 474
Dictionary Holder, Noyes' - - - - 474
Gratuitous Dentistry in French Schools, - 475
Transactions of Illinois State Dental Society, - 475
Cohesion of Molecules and their Resistance to Displacement Constituting Hardness, - - 30
Cohesive or Mechanical Force, - - - 26
Correspondence, - - - - - 30
Care of Teeth in Sickness, - - - - 92
Chromograph, - - - - - - 95
Charging for the "Know-how," - - - 96
Caldwell, J. J., M. D., - - - - - 65
Coyle, J. H., D. D. S., - - - - 78
Catlin, W. A. N., F. R. C S., - - - - 97
Chemistry and Therapeutics, - - - 223
Chemical and Physical Effects of Fillings upon Teeth, 231
Crude Blunders, - - - - - 236
Concerning a So-Called Dental College, - - 329
Coffin, Walter H. - - - - . - 385, 495
Causes of Irregularities of the Teeth, - - 392
Coles, Oakley - - - - - - 395
Concerning Dental Colleges, - - - - 420
Causes of Rapid Dental Decay, - - - 504
Causes of Death under Chloroform, - - 515
Correspondence—Stannous Gold, - 521
Civilization in its Relation to the Increased Degeneracy of the Human Teeth, - - - 458
Catching Cold—Remedies, - - - - 478
Cox, Ed. W., L. D. S., - - - - 180
Death from Nitrous Oxide, - - - - 335
Death from Ether, - - - - - 238
Dentists Constitutionally Treating Patients, - - 130
Dentistry in Paris, - - - - 85

Dentists for the Army and Navy, - - - 373
Dr. Clowes on Plastic Fillings, - - - 93
Drilling Glass, - - - - - - - 239
Disease of the Teeth in the International Medical
Congress, - - - - - - 385
Dental Surgery. The Study and Means thereto, - - 400
Death during Chloroform Inhalation, - - - 409
Denuding of Teeth, - - - - - - 512
Death under Chloroform, - - - - - 515
Dental Dealers' Convention, - - - - 517
Different Forms of Atrophy of the Teeth, - - 468
Durability of Gutta Percha Fillings, - - - 471
Dental Miscellany, Discontinuance of, - - - 471
Dangers of Bonwill's Method, - - - - 192
Editorial Notice, - - - - - 236
Endorsements, - - - - - - - 139
English Dentists Act. Imperfections of, - - 97
Extraction of Teeth followed by Insanity, - - 45
Extraordinary Nephritic Calculus, - - - 237
Fatty Tumor of the Tongue, - - - - 240
Female Doctors, - - - - - 40
Fillings, - - - - - - - - 481
Flagg, J. Foster, D. D. S., - - - - 420
Fletcher, Thomas, F. C. S., - - - - - 231
Food for Infants, - - - - - - 141
Fooled by Temperature, - - - - - - 236
Forty-second Annual Commencement of the Baltimore
College of Dental Surgery, - - - 518
Ford, W. W. Dr., - - - - - - - 223
Friedrichs, A. G., M. D , - - - - - 536
Georgia State Dental Society, - - - 210, 518
Gunning, Thos. B., D. D. S., - - - - - 392
Has Vaccination Anything to do with the Degeneration of
the Human Teeth? - - - - 74
Hardman, J. Thos., D. D. S.. - - - - 360
Holland's Dental Law, - - - - - 43
Hard on Dr. Atkinson, - - - - - 235
Heevenoid and Rubber Dam, - - - - - 90
Hackenberg, Dr. G. F. - - - - 515
Hardening and Tempering Intsruments, - - - 466
Hodgkin, Prof. Jas. B., D. D. S., - - - 348
Hunt, R. Finley, D. D S., - - - - 70
Hurtt, J. N., D. D. S., - - - - - 481
In Fine Company, - - - - - - - 91
Is Mercury Absorbed by the Skin? - - - 37
Ingersoll, L. C., D. D. S., - - - - - - 145
Irregularities of the Teeth. A General Treatment of, 385
Irregularities of Position of the Teeth, - - - 392

Irregularities. A Generalized Treatment of, - - 495
Keep Posted - - - - - - 238
Keeping Warm, - - - - - - 526
Kells, C. E., D. D. S., - - - - - 452
Kingsley, Norman, D. D. S., - - - - - 458
King, A., D. D. S., - - - - - 462
Largest Book Published, - - - - - 432
Lowry, Dr. M. B. - - - - - 553
Maryland and District of Columbia Dental Association,
Proceedings of Annual Meetings of 1881, 185, 337
McElhaney, G. W., D. D. S., - - - - - 512
Menthol, - - - - - - - 96
Merits of soft and Cohesive Gold as a Filling Material, 416
Mills, Geo. A., D. D. S., . - - - 26
Mississippi State Dental Association. Proceedings of, - 154
Mode of Origin of Some Secondary Lesions, - - 180
Marvin, C. A.. D. D. S., - - - - - 559
National Dental Association, - - - 134, 240
Nerve Regeneration, - - - - - 528
Neuralgia—Its Pathology and Etiology, - - 1
New Treatment of Abscesses, - - - - 431
Nitrous Oxide. - - - - - - 49
North Carolina Dental Association, - - 134, 241
Object and Treatment of Certain Irregularities
of the Teeth, - - - - 395
Odontalgia, - - - - - - 319
Odontological Society of Pennsylvania, - - 13
Oliver's Journal, - - - - - - - 531
Oral Electricity and the New Departure, - - 193
Omission, - - - - - - - 188
OBITUARY.
Dr. Byron F. Coy, - - - - - - 188
Prof E. Lloyd Howard, M. D., - - - 332
Dr. Daniel Harwood, - - - - 377
Dr. Joshua Tucker, - - - - 381, 472
Dr. Joseph S. Hartman, - - - - 382
Dr. M. S. Dean, - - - - 473, 522
M. Chas. Abbey, - - - - - 473
Oxygenation of Amalgam, - - - 480
Paralysis of the Facial Nerve, - - - 65
Patrick, John J. R., D. D. S., - - - - 193
Patents and the Medical Men, - - - 376
Parrot, M., - - - - - - - 468
Pease, W. A. Dr., - - - - - - 289
Pedley, F. N., D. D. S., - - - - - - 6
Plastic Fillings, - - - - - 93
Preliminary Prescribing, - - - - - 94

Preparatory Education, - - - - - 139
Preservation of the Teeth, - - - - 141
Peculiarites of Race, - - - - - 144
Peculiar Obstruction of Wharton's Duct, - - 144
Principal Causes of Constitutional Derangement of
First Dentition, - - - - - 360
Proceedings of Miss. State Dental Association, - - 154
Proceedings of Georgia State Dental Society, - 210
Proceedings of Southern and North Carolina
Dental Associations, - - - 241, 321
Pyorrhoea Alveolaris, - - - - 446
Rapid Breathing as a Pain Obtunder in Surgery, 111, 163
Rawls, A. O., D. D. S., - - - - 446
Relation of Tonsils to Virility, - - - - 96
Replanting of Teeth, - - - - - 78
Review of "Unfortunate Interference," - - - 318
Remarks on Proceedings of Southern Dental Association, 330
Restoring Partial Loss of Teeth without Plate, 175
Richardson, F., D. D. S., - - - - - 74
Rubber Question, - - - - - - 70
Rubber Patents, - - - - - - - 91
Salivary Calculus, - - - - - - 142
Syphilis about the Mouth, - - - - - 6
Structure of Metals, - - - - - . 38
Shumway, T. D., D. D S., - - - - 26
Sczinskey, T. S., M. D., - - - - 58
Sensitive Dentine, - - - - - - 190
Southern Dental Association, - 87, 134, 235, 241, 321
Stannous Gold, - - - - - - 521
Stebbing, Dr. E. A., - - - - - 423
Spalding, C. W., D. D. S., - - - - - 504
Stomatoscope, The, - - - - - - 187
Talbot, E. S., M. D., D. D. S., - - - - 433
The Chemistry and Physiological Action of Mercury
as used in Amalgam Fillings, - - - 433
The Teeth of the Rising Generation, - - 452
The Newest of Our Creeds, - - - - - 348
The Structure of Metals, - - - - - 38
The Rubber Question, - - - - - - 70
Therapy, - - - - - - - 140
Tempering Instruments, - - - - - - 466
To Prevent the Clouding of Mirrors by Moisture, - 46
To Restore India Rubber Goods, - - - 95
Townsend, H. H. Dr., - - - - - 302
Tomes, John, F. R. S., - - - - - - 400
Tongue Sucking as a Cause of the Protrusion
of the Front Teeth, - - - - - 462

The Opium Habit and Alcoholism, - - - 473
The New England Journal of Dentistry, - - 474
Tin Foil, - - - - - - 331
Tempering Steel, - - - - - 480
Treatment of Irregularities of the Teeth, - 385, 392
Treatment of the Dental Pulp, - - - - 366
Treatment of Dead and Dying Teeth, and
Alveolar Abscess, - - - - 302
Truman, James, D. D. S., - - - - 82, 123
The Stomatoscope, - - - - - 187
To Prevent the Clouding of Mirrors, - - - 190
Uniting Gold to Amalgam in Filling, - - - 475
Use of Platinum in Amalgam Alloys, - - - 477
Underwood, Arthur S., D. D. S., Etc., - - - 546
Vanderbilt University, Dental Department, - - 521
Wanted, - - - - - - - 40
West Virginia Dental Law, - - - 36
Watt, Geo., M. D., D. D. S., - - - - - 49
Wilson, S. S., M. D., - - - - 1
Washington City Dental Society, - - - - 429
Williams, G. J., D. D. S., - - - - 562
Why there is a Period to Life, - - - - 58
Women as Physicians, - - - - 335

*Wanted—Copies of this Journal.*—Dr. George H. Perine, 116 West 42nd St., New York City, wishes to obtain volumes 1, 2, 3, 4, 5 and 6 of the *first series* of this Journal, and also volumes 5 and 6 of the *third* series. Any one wishing to dispose of such will confer a favor on Dr. P., by corresponding with him.

### To Readers and Correspondents.

Communications intended for publication, and exchange journals and papers, should be addressed to the EDITOR OF THE AMERICAN JOURNAL OF DENTAL SCIENCE, 259 N. Eutaw St., Baltimore, Md. All letters relating to the business of the journal, or containing cash remittances, to be directed to Messrs. SNOWDEN & COWMAN. Articles for publication should be received by the editor at least three weeks previous to the first of the month on which the number in which it is designed for them to appear, is issued.

### CONTENTS:

MONTHLY SUMMARY.

Holland's Dental Law .....43

Extraction of Teeth followed by Insanity .....45

To Prevent the Clouding of Mirrors by Moisture .....46

A Case of Fracture of the Teeth Producing Severe Symptoms .....47

Alcohol a Stimulant or Narcotic .....48

☞The American Journal of Dental Science will contain fifty pages of reading matter in each number, making a volume of about

*Six Hundred* pages,

EXCLUSIVE OF ADVERTISEMENTS.

THE

### AMERICAN JOURNAL OF DENTAL SCIENCE.

The *Journal* is issued on the first of each month and contains not less than *fifty* pages of reading matter in each number, exclusive of advertisements, making a volume of *six hundred* pages per annum.

In each number will be found Original Communications, Correspondence, Selected Articles, Monthly Summary, Bibliographical Notices and Editorial Matter, and every effort will be exerted to render the *Journal* worthy of the patronage of the Dental profession.

A generous co-operation is respectfully solicited from the members of the profession; and as the *Journal* has now a printing office of its own, which materially lessens the cost of its publication, it will be issued at the reduced subscription price of

**$2.50 Per Annum in Advance.**

Communications are respectfully solicited on all subjects pertaining to the practice of dentistry, as well as reports of cases occurring in dental practice.

### RATES OF ADVERTISING.

| | 1 mo. | 2 mo. | 3 mo. | 6 mo. | 12 mo. |
|---|---|---|---|---|---|
| 1 Page. | $15 00 | $22 50 | $30 00 | $50 00 | $100 00 |
| ½ " | 10 00 | 15 00 | 20 00 | 30 00 | 50 00 |
| ¼ " | 7 00 | 11 00 | 15 00 | 20 00 | 30 00 |
| ⅛ " | 5 00 | 8 00 | 11 00 | 15 00 | 20 00 |

All original communications designed for publication, works for review, new instruments for notice, exchanges, &c., should be directed to the editor, 259 N. Eutaw St., Baltimore, Md.

All advertisements and subscriptions should be addressed to

SNOWDEN & COWMAN,

*Publishers of the Am. Journal of Dental Science.*

86 W. Fayette St. Bet. Charles & Liberty Sts.,

BALTIMORE, MD.

